# Hypoxia Activates Notch4 via ERK/JNK/P38 MAPK Signaling Pathways to Promote Lung Adenocarcinoma Progression and Metastasis

**DOI:** 10.3389/fcell.2021.780121

**Published:** 2021-12-20

**Authors:** Xiaochen Li, Xiaopei Cao, Hanqiu Zhao, Mingzhou Guo, Xiaoyu Fang, Ke Li, Lu Qin, Yuanzhou He, Xiansheng Liu

**Affiliations:** ^1^ Department of Pulmonary and Critical Care Medicine, Tongji Hospital, Tongji Medical College, Huazhong University of Science and Technology, Wuhan, China; ^2^ Key Laboratory of Respiratory Diseases, National Ministry of Health of the People’s Republic of China and National Clinical Research Center for Respiratory Disease, Wuhan, China; ^3^ Department of Pediatrics, Tongji Hospital, Tongji Medical College, Huazhong University of Science and Technology, Wuhan, China

**Keywords:** lung adenocarcinoma, hypoxia, Notch4, mitogen-activated protein kinase, proliferation, apoptosis, migration

## Abstract

Hypoxia contributes to the progression and metastasis of lung adenocarcinoma (LUAD). However, the specific underlying molecular mechanisms have not been fully elucidated. Here we report that Notch4 is upregulated in lung tissue from lung cancer patients. Functionally, Hypoxia activates the expressions of *Delta-like 4* and Notch4, resulting in the excessive proliferation and migration of LUAD cells as well as apoptotic resistance. Notch4 silencing reduced ERK, JNK, and P38 activation. Meanwhile, Notch4 overexpression enhanced ERK, JNK, and P38 activation in LUAD cells. Furthermore, Notch4 exerted pro-proliferation, anti-apoptosis and pro-migration effects on LUAD cells that were partly reversed by the inhibitors of ERK, JNK, and p38. The binding interaction between Notch4 and ERK/JNK/P38 were confirmed by the co-immunoprecipitation assay. *In vivo* study revealed that Notch4 played a key role in the growth and metastasis of LUAD using two xenograft models. This study demonstrates that hypoxia activates Notch4-ERK/JNK/P38 MAPK signaling pathways to promote LUAD cell progression and metastasis.

## Introduction

Lung cancer is the most common cancer and the leading cause of cancer death in both men and women worldwide (Siegel et al., 2018). Lung adenocarcinoma (LUAD) accounts for 40% of all types of lung cancer (Imielinski et al., 2012). Lung cancer is characterized by sustained cell proliferation, resistance to cell death, invasion and metastasis (Hanahan and Weinberg, 2011). Intratumoral hypoxia is a critical microenvironmental factor driving cancer progression and is associated with poor clinical prognosis (Taiakina et al., 2014; Schito and Semenza, 2016; Li et al., 2021). Hypoxia upregulates a large number of oncogenes that contribute to the excessive proliferation, invasion, metastasis, and so on (Wigerup et al., 2016; Mennerich et al., 2019). However, the underlying molecular mechanisms of hypoxia involved in LUAD cell progression and metastasis remain largely unknown.

The Notch signaling pathway is an evolutionarily conserved pathway that is important for cell fate and behavior during embryogenesis (Artavanis-Tsakonas et al., 1999). In mammals, the Notch receptor family consists of four single transmembrane receptors (Notch1-4) that share a similar structure and five membrane-bound ligands (Delta-like1/3/4 and Jagged1/2) (Niessen and Karsan, 2008). Upon local receptor-ligand interaction, Notch receptor intracellular domain (ICD) is released into the cytoplasm by sequential proteolytic cleavage, which transduces Notch signaling and regulates cell differentiation, proliferation, and apoptosis (Siebel and Lendahl, 2017). Previous studies suggest hypoxia may activate Notch signaling pathway (Jögi et al., 2002), implying that the Notch signaling pathway is an attractive candidate mediator between hypoxia and cancer. Recent studies show that Notch4 acts as an oncogene in some types of cancers, such as colorectal cancer (Wu et al., 2018), triple-negative breast cancer (Zhou et al., 2020), and prostate cancer (Zhang et al., 2017). However, the role of Notch4 in hypoxic LUAD and the underlying mechanism is unclear.

HEY and HES are targets of canonical Notch signaling, which relies on the translocation of ICD into the nucleus where it binds to co-activator proteins and forms a nuclear transcriptional activator complex formation (De Strooper et al., 1999; Struhl and Greenwald, 2001; Francis et al., 2002; Hu et al., 2002). On the other hand, Notch non-canonically exerts its biological functions via the crosstalk with mitogen-activated protein kinase (MAPK) signaling in cell cytoplasm (Kiec-Wilk et al., 2010). MAPK families, including ERK, JNK, and P38 kinases (Wei et al., 2010; Kim and Choi, 2015), play a critical role in a broad spectrum of tumorigenesis and development (Braicu et al., 2019). This study aims to investigate the role of Notch4 in hypoxic LUAD and underlying molecular mechanism both *in vitro* and *in vivo*, respectively.

## Materials and Methods

### Tissue Samples

LUAD tissues and adjacent non-malignant lung tissues were collected from Tongji Hospital of Huazhong University of Science and Technology (Wuhan, China). Ethical approval was obtained from the Ethics Committee of Tongji Hospital and written informed consent was obtained from each patient. All animal experiments were performed in compliance with the guidelines for animal testing and research, with ethical approval from Tongji Hospital. (Wuhan, China; Approval number: TJH-202011004).

### Cell Lines and Cultures

Human cell lines A549 and human bronchial epithelial (HBE) were from ATCC, and H1299 was obtained from the Institute of Biochemistry and Cell Biology of the Chinese Academy of Sciences. The normal HBE cells are used as negative control representing non-cancerous cells (Byun et al., 2013). All cells were cultured in Roswell Park Memorial Institute-1640 medium supplemented with 10% fetal bovine serum and maintained in a 5% CO_2_ incubator at 37°C. The hypoxic cells were cultured in a 2% O_2_ incubator (Galaxy R; RS Bitotech, Alloa, UK) continually gassed with 5% CO_2_ and 93% N_2_ as previously described (Cao et al., 2020).

### Antibodies and Chemicals

Primary antibody against B cell leukemia/lymphoma 2 (Bcl-2) was obtained from Boster (Wuhan, Hubei, China). Primary antibodies against polyclonal antibodies against proliferating cell nuclear antigen (PCNA), Bcl-2 associated X (Bax), survivin, matrix metallopeptidase 9 (MMP9), matrix metallopeptidase 2 (MMP2), β-Actin and normal rabbit or mouse immunoglobulin G (IgG) were obtained from Proteintech (Wuhan, Hubei, China). Primary antibody against Notch4 was obtained from Santa Cruz Biotechnology (Dallas, TX, United States). Primary antibodies against P38/p-P38 MAPK, JNK/p-JNK, ERK/p-ERK were obtained from Cell Signaling Technology (Danvers, MA, United States). HRP-conjugated anti-Rabbit IgG and HRP-conjugated anti-mouse IgG were obtained from Servicebio (Wuhan, Hubei, China). HRP-conjugated anti-Rabbit IgG light chain and HRP-conjugated anti-mouse IgG light chain were obtained from Abbkine Scientific (Redlands, CA, United States). Protein G magnetic beads were obtained from Cell Signaling Technology (Danvers, MA, United States). U0126, SP600125 and SB203580 were obtained from MedChemExpress (Monmouth Junction, NJ, United States).

### Cell Transfection

Small interfering RNAs against Notch4 and negative control siRNA were synthesized by RiboBio (Guangzhou, China) and transfected into cells using Lipofectamine 3000 (Invitrogen, Carlsbad, CA, United States) according to the manufacture’s instruction. The siRNA sequence was as follows: CAA​CGG​GCA​CTG​TGA​GAA​A. When reaching 40–60% of confluence, the cells were transfected with 50 nmol siRNA using Lipofectamine 3000 (Invitrogen, Carlsbad, CA, United States). Notch4 overexpression plasmid and negative control plasmid were purchased from GeneChem (Shanghai, China). The mammalian expression plasmid for Flag-tagged Notch4 was constructed by molecular cloning and confirmed by DNA sequence. When reaching 80–90% of confluence, the cells were transfected with 1.5 μg purified plasmid. Finally, the mRNA and protein levels of cells were analyzed by quantitative real-time PCR and western blotting at 48 and 72 h after transfection, respectively.

Lentivirus was produced by Hanbio Biotechnology Corporation. To established stable cell lines, the lentivirus stocks were used to transduce A549 cells. After 48 h post-transduction, the cells were maintained in RPMI-1640 medium containing 1 μg/ml puromycin for at least 7 days. Finally, the mRNA and protein levels of cells were analyzed by quantitative real-time PCR and western blotting. The target sequences for Notch4 were as follows: shNotch4-1: 5′-GCT​CTG​GAA​AGA​GGG​TTT​AAG-3′; shNotch4-2: 5′-ACA​ACG​GGC​ACT​GTG​AGA​AAG-3′; shNotch4-3: 5′-CGA​TAA​AGA​TGC​CCA​GGA​CAA-3′.

### Real-Time Quantitative PCR

Total RNA was extracted using Trizol (Takara, Dalian, China) according to the manufacture’s instruction. RNA concentration was determined by Nanodrop analysis. Then 500 ng of RNA was used to synthesize cDNA using PrimeScript RT reagent kit (Takara, Dalian, China). Quantitative RT-PCR was performed using SYBR Green Mix (Takara, Dalian, China). The primer sequences were as follows: Notch4 forward, 5′-CGT​ACC​CCA​CTT​CAC​ACT​GC-3′, reverse, 5′-AGG​TGT​AGT​CCC​GTC​GTC​TG-3′; *Delta-like 4* forward, 5′-CAC​CTG​CTA​CAC​CGA​CCT​CTC​C-3′, reverse, 5′-TCC​GAC​AAG​TTG​TTC​ATG​GCT​TCC-3′.

The cycling conditions were as follows: initial denaturation for 10 min at 95°C followed by 40 cycles of denaturation (15 s at 95°C), annealing and elongation (30 s at 60°C). The relative expression of gene was calculated using 2^−ΔΔCt^ method using β-Actin as the reference gene.

### Western Blotting Analysis

Total cellular protein was extracted by RIPA lysis (50 mM Tris, 150 mM NaCl, 1% NP-40, 0.5% sodium deoxycholate, 0.1% SDS, PH7.4) buffer supplemented with phenylmethylsulfonyl fluoride, cocktail, and phosphorylation protease inhibitor. Cell lysis was centrifuged at 12,000 rpm for 15 min, then the supernatants were collected for determination of the protein concentration by BCA assay. All steps were performed at 4°C. 20 μg of protein were subjected to 10% sodium dodecyl sulfate–polyacrylamide gel electrophoresis followed by western blotting. The signals were detected with a chemiluminescent substrate system (Bio-Rad, Hercules, CA, United States). Relative expressions of target proteins were quantified by Image J software.

### Cell Viability Assay

Cell viability was measured using the Cell Counting Kit-8 assay according to the manufacture’s instruction. The cells were seeded into 96-well plates at the cell density of 3,000 cells per well for 24 h. Then the medium was replaced with different transfection mixture. After 6 h incubation, the transfection mixture was replaced with medium supplemented with 10% fetal bovine serum, and the cells were further incubated for another 24 h. After that, cells were incubated under normoxia or hypoxia for 24 h. Finally, 10 μL CCK8 (Cell Counting Kit-8, Dojindo, Japan) reagent was added into per well and determined by ELx800 Universal Microplate Reader (Bio-Tek Instruments, Inc., Winooski, VT, United States).

### Edu Proliferation Assay

The cells were seeded into 96-well plates (3,000 cells per well) and performed according to protocols mentioned above. Edu Cell Proliferation Assay Kit (RiboBio, Cell-Light™EdU Apollo^®^643 *In Vitro* imaging kit) was used to assessed cell proliferation according to the manufacture’s instruction. The cells were added with 100 μL Edu (diluent reagent A with a complete medium by 1:1,000) and incubated for 2 h at 37°C. The cells were fixed in 4% paraformaldehyde for 15–30 min and incubated with 1× Apollo^®^ solution for 30 min at room temperature. The cell nuclei were stained with 100 μL 1× Hoechst33342 for 30 min. Finally, the cells were examined under a fluorescent microscope (Olympus, Japan). Data were presented as a fold-change of Edu-incorporating cells compared with negative controls.

### Colony Formation Assay

The transfected cells were seeded into a 6-well plate (200–400 cells per well) and cultured for 7–14 days. After incubation, cell colonies were fixed with 4% paraformaldehyde for 15 min, then stained with 1% crystal violet. The numbers of colonies were counted under a microscope.

### Cell Apoptosis Assay

The cells were seeded into a 6-well plate (100,000 cells per well). Firstly, cells were starved in serum-free for 24 h. After transfection for 48 h, cells were exposed to normoxia or hypoxia for 24 h. Then cells were collected and resuspended in 200 μL binding buffer per well. The cells were labeled with 5 μL Annexin-V and propidium iodide (PI) to assess cell apoptosis using an Annexin-V/PI detection kit (Keygen Biotech, Nanjing, China). Finally, apoptotic cells were analyzed using flow cytometry (BD Biosciences, San Jose, CA, United States). Apoptotic cells including early apoptotic cells (Annexin-V positive and PI-negative) and late apoptotic cells (Annexin-V positive and PI-positive) were shown.

### Cell Migration Assay

Cell migration assay was performed in a 24-well plate (Corning, MA, United States) according to the manufacture’s instruction. Firstly, 200 μL cell suspensions (10,000 cells totally) containing 1% fetal bovine serum were seeded into the upper chamber, while 600 μL medium containing 15% fetal bovine serum was added into lower chambers. The cells were cultured under normoxia or hypoxia for 24 h. After incubation, cells on the lower surface of the membrane were fixed with 4% paraformaldehyde and stained with 0.1% crystal violet. Non-migrated cells were removed by scraping the membranes with a cotton swab from the upper surface. Finally, cells were counted under an optical microscope (Zeiss, Oberkochen, Germany).

### Wound Healing Assay

Wound healing assay were performed through creating a gap in confluent monolayer of cells using a pipette tip. The cell culture dish should be placed under an optical microscope (Zeiss, Oberkochen, Germany) to acquire image at 0, 24 h after the scratch, respectively. Photographs of 5 random fields were recorded for quantification analysis. Wound width was calculated as the average distance between the edges of the scratches.

### Immunoprecipitation

Co-immunoprecipitation experiments were performed according to the manufacturer’s protocol. Briefly, cells were collected and lysed in IP-lysis buffer (50 mM Tris, 150 mM NaCl, 1% NP-40) and protease inhibitor. Supernatants were collected by centrifugation (14,000 rpm, 10 min, 4°C), and pre-cleared with 20 µL Protein G Magnetic beads (Cell Signaling Technology) for 2 h. Then the pre-cleared supernatants were incubated with the indicated antibodies (2 μg/ml) overnight at 4°C, followed by immunoprecipitation with 20 µL pre-washed Protein G Magnetic beads for 3 h at 4°C. Finally, the precipitates were washed 5–7 times with IP-lysis buffer and detected using Western blotting.

### Animal Experiments

Male BALB/c nude mice (3–4 weeks) were purchased from Charles River Company. All animal experiments were performed in compliance with the guidelines for animal testing and research, with ethical approval from Tongji Hospital. A subcutaneous tumor xenograft model and a tail vein model were used to evaluate xenograft tumor growth and metastasis *in vivo,* respectively. For the subcutaneous tumor growth model, stable A549 cells (1 ⨯ 10^6^) treated with shNotch4 or scramble control were subcutaneously injected into the right dorsal flank of 5-week-old male BALB/c nude mice. Tumor volume (TV) was measured by vernier caliper and calculated as following: TV (mm^3^) = (L ⨯ W^2^)/2 (L, long diameter; W, wide diameter). Tumors were separated from sacrificed mice, and then snap-frozen in liquid nitrogen or fixed for hematoxylin-eosin staining. For the tail vein model, stable cells (1 ⨯ 10^6^) were injected into the tail vein of 5-week-old male BALB/c nude mice. All the mice were sacrificed 6–8 weeks after injection. The lungs were separated from sacrificed mice and fixed for hematoxylin-eosin staining.

### Statistical Analysis

Statistical analyses were performed using GraphPad Prism software (Version 8.0). The student’s t-test was used to compare two groups. One-way analysis of variance was used to compare more than two groups, followed by Tukey’s multiple comparison test. Data were presented as means ± SD. *p* < 0.05 was considered statistically significant.

## Results

### Notch4 Expression Is Upregulated in LUAD

**FIGURE 1:**
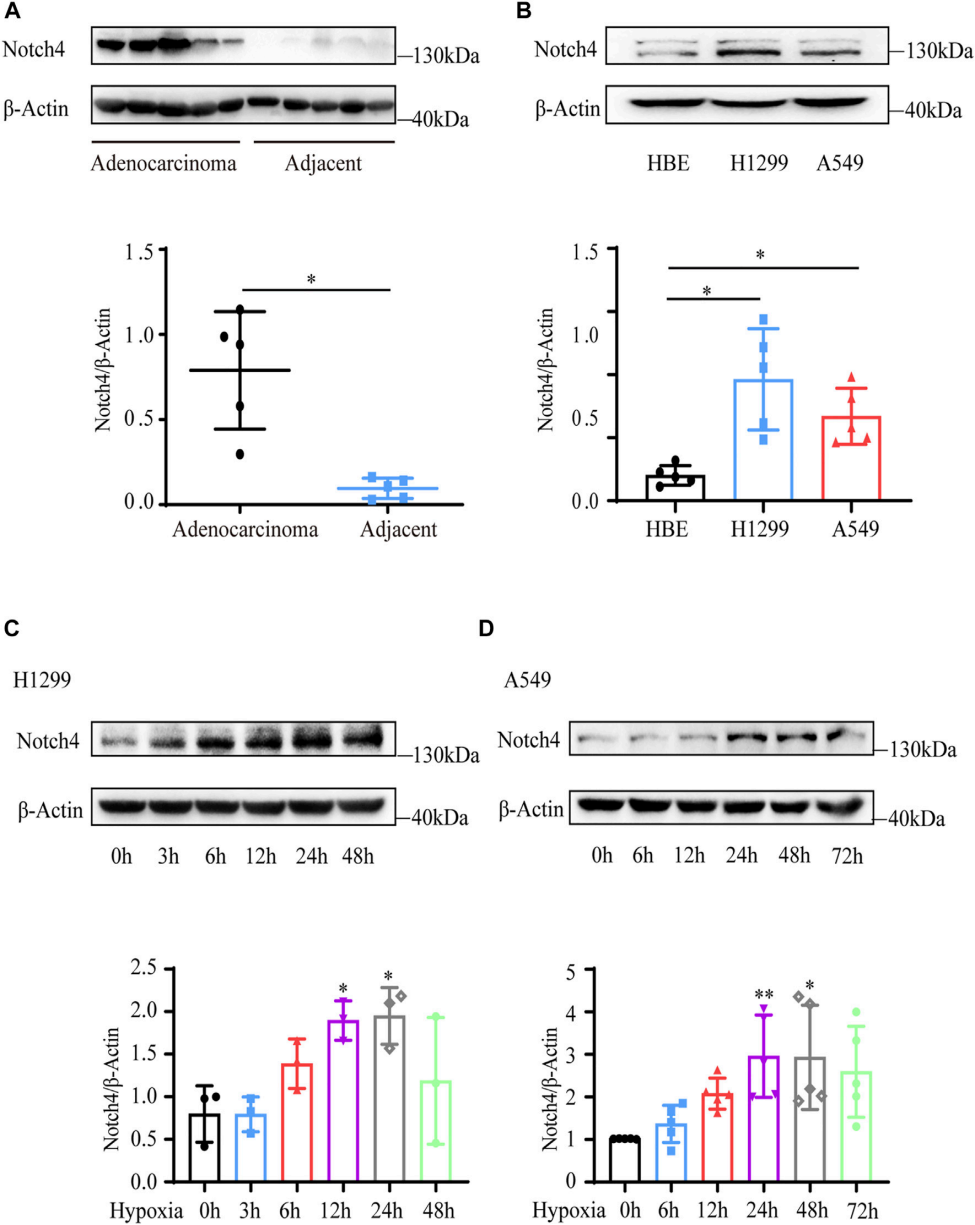
Notch4 is upregulated in LUAD tissues and cells. **(A)** The protein level of Notch4 in five paired LUAD tissues and adjacent non-malignant tissues. **(B)** The protein levels of Notch4 in A549 and H1299 cells and normal HBE cell. (*n* = 8). **(C)** The protein levels of Notch4 in H1299 cells exposed to hypoxia for different times. (*n* = 3). **(D)** The protein levels of Notch4 in A549 cells exposed to hypoxia for different times. (*n* = 5). Data were presented as means ± SD. *p < 0.05, **p < 0.01. LUAD, lung adenocarcinoma; HBE, human bronchial epithelial.

Five paired LUAD tissues and adjacent normal tissues were collected from patients who underwent lung resection. The protein level of Notch4 was increased in LUAD tissues compared with the adjacent normal tissues (Figure 1A). The expressions of Notch4 were significantly increased in A549 and H1299 cells compared with that observed in HBE cells (Figure 1B). We further assessed Notch4 expression in A549 and H1299 cells exposed to hypoxia for different times given the central role of hypoxia in the regulation of tumor progression. The protein levels of HIF-1a were significantly increased in A549 and H1299 cells under hypoxia over time, indicating a successful induction of hypoxic condition (Supplementary Figures S1A,B). Our results revealed that hypoxia significantly enhanced Notch4 protein level over time reaching a peak after 24 h (Figures 1C,D). Meanwhile, increased mRNA level of Notch4 and *Delta-like 4* were also observed in H1299 cells exposed to hypoxia (Supplementary Figures S1C,D), which may indicate that hypoxia activates Notch4 signaling by increasing the levels of *Delta-like 4*.

### Notch4 Promotes Xenograft Tumor Growth and Metastasis *In Vivo*

**FIGURE 2:**
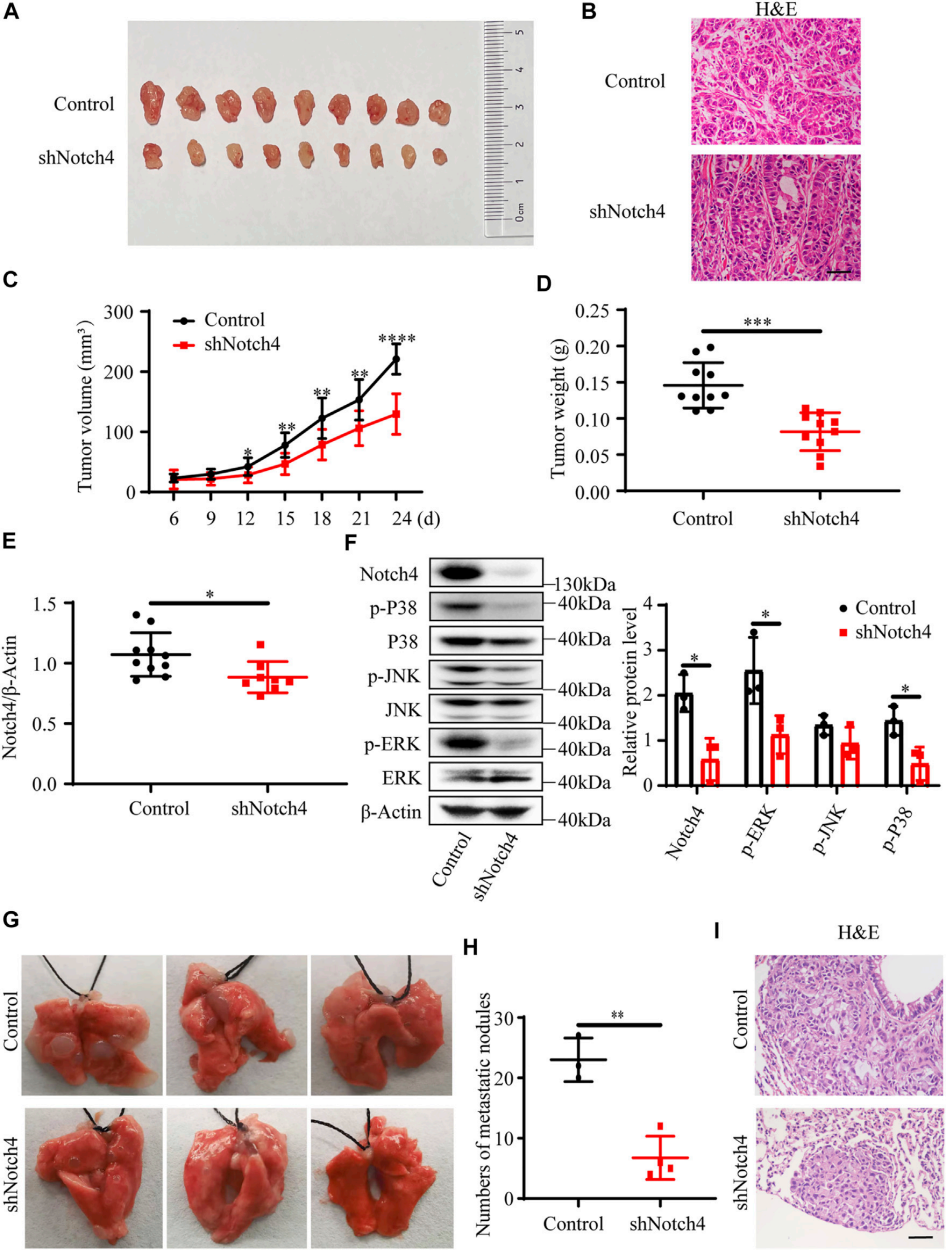
Notch4 gene silencing alleviated xenograft tumor growth and metastasis. **(A–F)** Xenograft tumor growth experiments were performed in nude mice with A549-control and A549-shNotch4 stable cells. **(A)** Representative images of subcutaneous tumor dissected from the nude mice were presented. **(B)** Representative H&E staining images in sections of tumor were presented. Magnification, ×200; Bar, 50 μm. **(C)** Subcutaneous tumor growth curves of the nude mice were presented. (*n* = 10). **(D)** Subcutaneous tumor weights were presented. (*n* = 10). **(E)** The mRNA level of Notch4 in xenograft tumor. (*n* = 8–10). **(F)** The protein levels of Notch4, p38, p-P38, JNK, p-JNK, ERK, p-ERK in xenograft tumor. (*n* = 3). **(G, I)** Lung metastasis experiments were performed in nude mice with A549-control and A549-shNotch4 stable cells. **(G)** Representative images of lung metastases were presented. **(H)** The numbers of visible metastatic nodules in the lungs of mice were counted. (*n* = 3–4). **(I)** Representative H&E staining images in sections of lung tissues were presented. Magnification, ×200; Bar, 50 μm. Data were presented as means ± SD. *p < 0.05, **p < 0.01, ***p < 0.001, ****p < 0.0001. H&E, hematoxylin-eosin staining. JNK, c-Jun N-terminal kinase; ERK, extracellular signal-regulated kinase.

A subcutaneous tumor xenograft model and a tail vein model were used to evaluate xenograft tumor growth and metastasis *in vivo*, respectively. Our results showed that a significant reduction in tumor weight and in tumor volume was observed in tumor originating from shNotch4-treated A549 cells compared with scramble control (Figures 2A–D). Quantitative RT-PCR revealed a decreased Notch4 mRNA level in shNotch4-treated xenograft tumors relative to matched controls (Figure 2E). Western blotting analysis suggested that shNotch4-treated tumors had lower levels of phosphorylated ERK, JNK, and P38 than controls (Figure 2F). Additionally, Notch4 knockdown in A549 cells significantly reduced metastatic growth in lungs compared to controls (Figures 2G–I). Above all, these results demonstrate that silencing of Notch4 inhibits tumor progression and metastasis of LUAD cells *in vivo*. Meanwhile, the tumors originating from A549 cells treated with Notch4 overexpression plasmid grew more rapidly than controls (Supplementary Figures S2A–C). Increased levels of phosphorylated ERK, JNK, and P38 were observed in tumor with Notch4 overexpression compared with negative controls (Supplementary Figure S2F). Metastatic spread of Notch4-overexpressing A549 cells were faster than controls (Supplementary Figures S2G–I). Based on these above results, we conclude that Notch4 facilitates the growth and metastasis of LUAD and ERK/JNK/P38 pathways may be involved in the process *in vivo*.

### Notch4 Regulates LUAD Cells Proliferation, Apoptosis, and Migration Under Hypoxia

**FIGURE 3:**
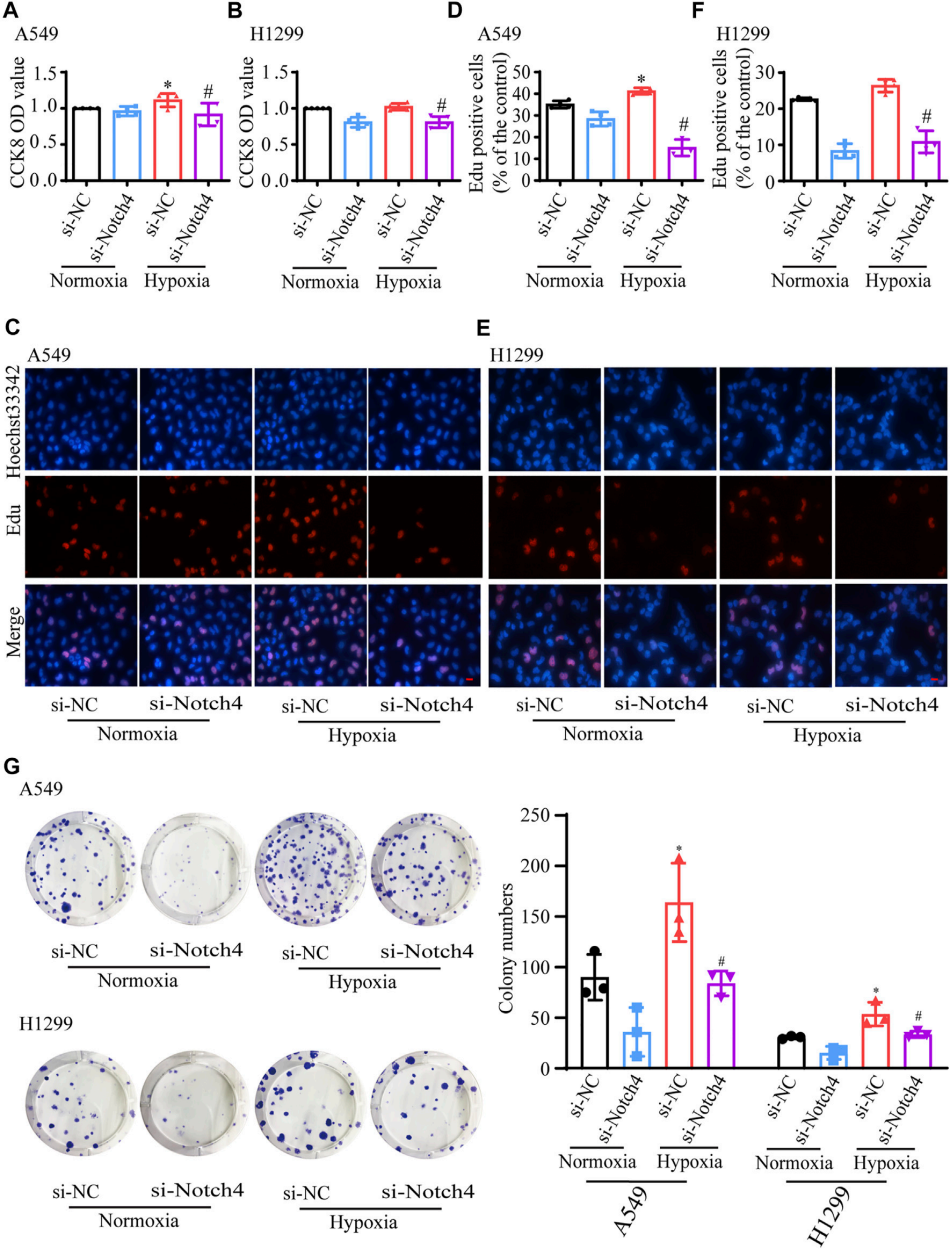
Notch4 is involved in the regulation of cell proliferation in A549 and H1299 cells exposed to hypoxia. **(A, B)** Cell viability was assessed using CCK-8 assay in A549 and H1299 cells (*n* = 4–5). **(C, D)** A549 cell proliferation was assessed using Edu assay. (*n* = 4). Magnification, ×400; Bar, 20 μm. **(E, F)** H1299 cell proliferation was assessed using Edu assay. (*n* = 5). Magnification, ×400; Bar, 20 μm. **(G)** Colony formation assay was performed in A549 and H1299 cells. (*n* = 3). Data were presented as means ± SD. *p < 0.05, comparison with normoxic cells treated with si-NC; ^#^ *p* < 0.05, comparison with hypoxic cells treated with si-NC. CCK-8, Cell Counting Kit-8; si-NC, negative control short interfering RNAs (siRNA); si-Notch4, the siRNA against Notch4.

**FIGURE 4:**
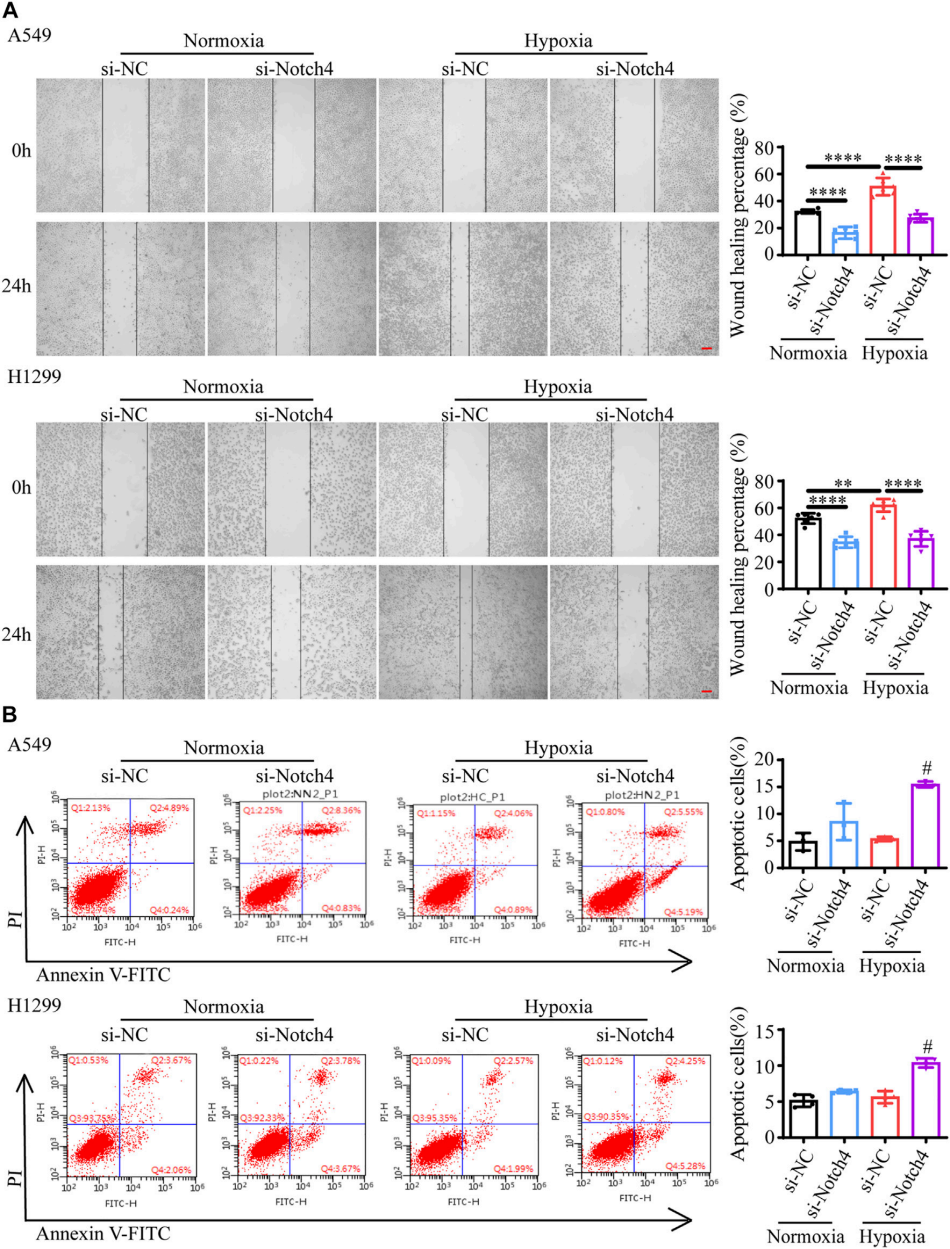
Notch4 is involved in the regulation of cell migration and apoptosis in A549 and H1299 cells exposed to hypoxia. **(A)** Wound healing assay was performed in A549 and H1299 cells. (*n* = 4). Magnification, ×40; Bar, 200 μm. **(B)** Cell apoptosis was assessed by Annexin-V/PI staining. (*n* = 3). Analyses of apoptosis including early apoptosis (Annexin-V positive and PI negative) and late apoptosis (Annexin-V positive and PI positive) were shown. Data were presented as means ± SD. *p < 0.05, **p < 0.01, ****p < 0.0001, comparison with normoxic cells treated with si-NC; ^#^ *p* < 0.05, comparison with hypoxic cells treated with si-NC. si-NC, negative control short interfering RNAs (siRNA); si-Notch4, the siRNA against Notch4; PI, propidium iodide.

The protein levels of Notch4 were significantly reduced in A549 and H1299 cells treated with siRNA against Notch4 relative to negative controls (Supplementary Figure S3). Hypoxia promoted the proliferation and migration of A549 and H1299 cells, and inhibited cell apoptosis. The knockdown of Notch4 partly abolished the excessive cell proliferation, migration and apoptosis resistance due to hypoxia compared with control using Cell Counting Kit-8, Edu staining, colony forming assay, wound healing assay, and Annexin-V/PI staining (Figures 3, 4). In addition, the knockdown of Notch4 significantly downregulated the protein expressions of survivin and MMP9, and upregulated the ratio of Bax/Bcl-2 (Supplementary Figure S4). Taken together, these findings indicate that Notch4 promotes A549 and H1299 cells proliferation and migration, and inhibits cell apoptosis under hypoxia.

### ERK, JNK, and P38 MAPK Signaling Pathways Mediate the Regulation of Notch4 Overexpression on A549 and H1299 Cells Proliferation, Apoptosis, and Migration

**FIGURE 5:**
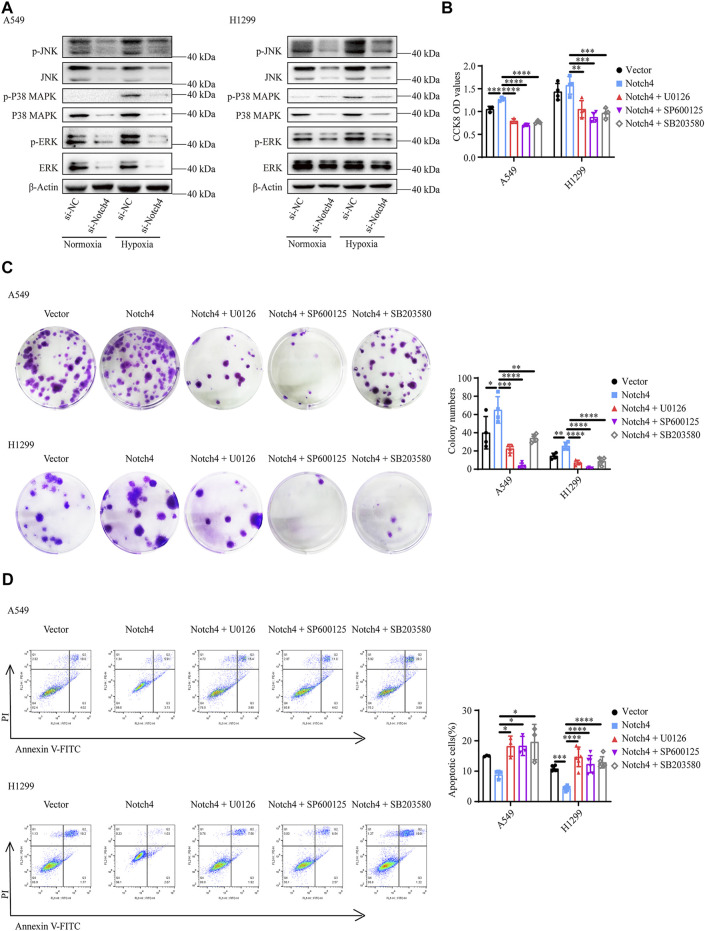
ERK, JNK, and P38 MAPK signaling mediate the regulation of Notch4 overexpression on A549 and H1299 cells proliferation and apoptosis. **(A)** The MAPK signaling pathway were detected in A549 and H1299 cells transfected with siRNA against Notch4 or negative control siRNA. **(B)** Cell viability were examined in A549 and H1299 cells transfected with Notch4 plasmid or negative control plasmid co-treatment with ERK (U0126), JNK (SP600125), or P38 (SB203580) MAPK pathway inhibitors by using CCK-8 assay. (*n* = 3–4). **(C)** Colony formation assay was performed in A549 and H1299 cells transfected with Notch4 plasmid or negative control plasmid co-treatment with U0126, SP600125, or SB203580 for 24 h (*n* = 4). **(D)** The Annexin-V/PI assay was performed in A549 and H1299 cells transfected with Notch4 plasmid or negative control plasmid co-treatment with U0126, SP600125, or SB203580 for 24 h (*n* = 3–6). Analyses of apoptosis including early apoptosis (Annexin-V positive and PI negative) and late apoptosis (Annexin-V positive and PI positive) were shown. Data were presented as means ± SD. *p < 0.05, **p < 0.01, ***p < 0.001, ****p < 0.0001. CCK-8, Cell Counting Kit-8; PI, propidium iodide; si-NC, negative control short interfering RNAs (siRNA); si-Notch4, the siRNA against Notch4. Vector, negative control plasmid; Notch4, Notch4 plasmid. ERK, extracellular signal-regulated kinase; JNK, c-Jun N-terminal kinase; MAPK, mitogen-activated protein kinase.

**FIGURE 6:**
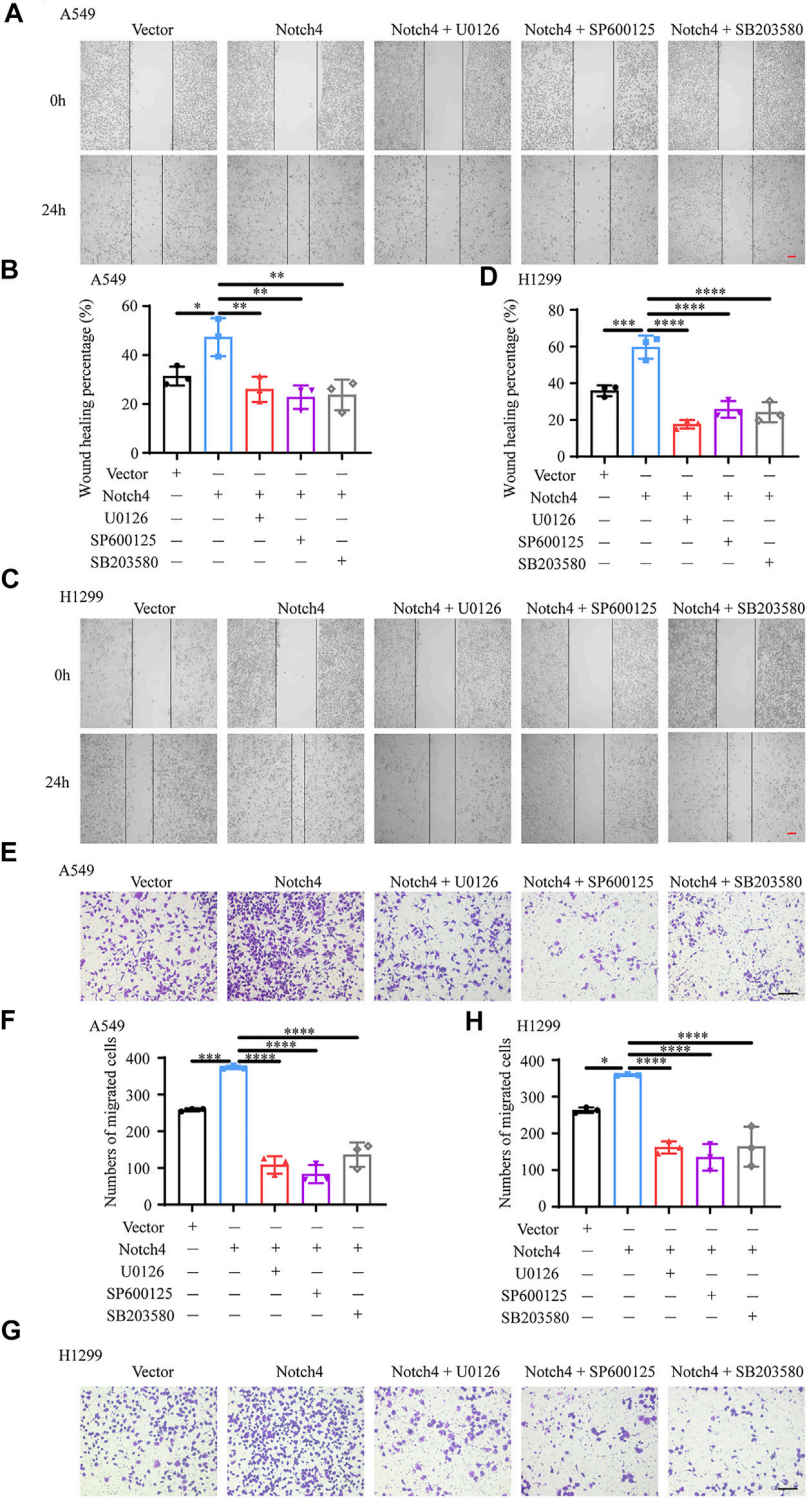
ERK, JNK, and P38 MAPK signaling mediate the regulation of Notch4 overexpression on A549 and H1299 cells migration. **(A–D)** Wound healing assay was performed in A549 and H1299 cells transfected with Notch4 plasmid or negative control plasmid co-treatment with U0126, SP600125, or SB203580. (*n* = 3). Magnification, ×40; Bar, 200 μm. **(E–H)** Transwell migration assay was performed in A549 and H1299 cells transfected with Notch4 plasmid or negative control plasmid co-treatment with U0126, SP600125, or SB203580 for 24 h (*n* = 3). Magnification, ×100; Bar, 50 μm. Data were presented as means ± SD. *p < 0.05, **p < 0.01, ***p < 0.001, ****p < 0.0001. Vector, negative control plasmid; Notch4, Notch4 plasmid. ERK, extracellular signal-regulated kinase; JNK, c-Jun N-terminal kinase; MAPK, mitogen-activated protein kinase.

**FIGURE 7:**
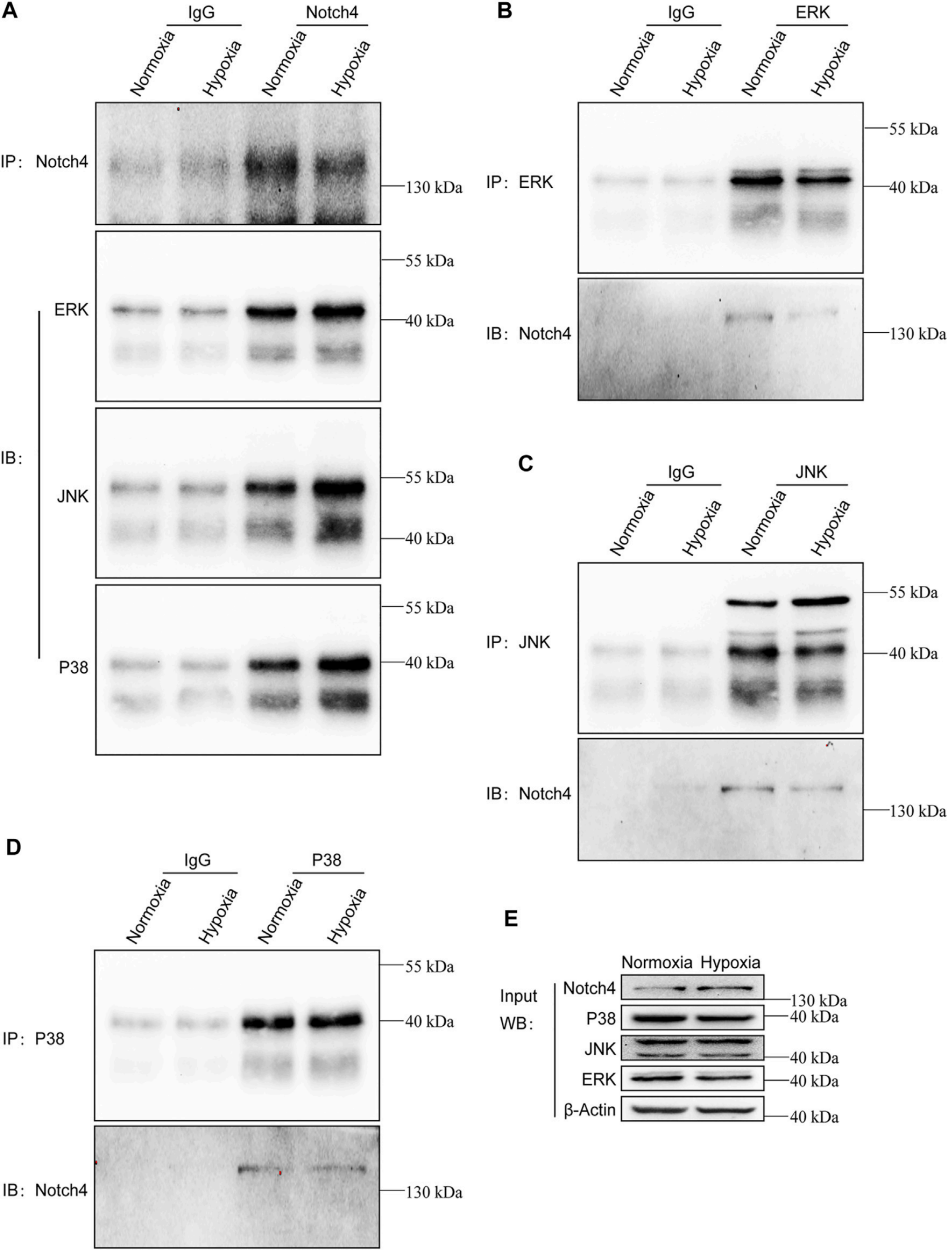
Notch4 interacts with ERK, JNK, and P38 in A549 cells. Endogenous co-immunoprecipitation was performed in A549 cells exposed to normoxia or hypoxia for 24 h. **(A)** Western blot analysis for endogenous ERK, JNK, and P38 after IP of endogenous Notch4 from A549 cells exposed to normoxia or hypoxia. **(B)** Western blot analysis for endogenous Notch4 after IP of endogenous ERK from A549 cells exposed to normoxia or hypoxia. **(C)** Western blot analysis for endogenous Notch4 after IP of endogenous JNK from A549 cells exposed to normoxia or hypoxia. **(D)** Western blot analysis for endogenous Notch4 after IP of endogenous P38 from A549 cells exposed to normoxia or hypoxia. **(E)** Whole-cell lysates were used for IB with the indicated antibodies to show expression. IP, immunoprecipitation; IB, immunoblot. ERK, extracellular signal-regulated kinase; JNK, c-Jun N-terminal kinase.

The activity of MAPK family members including ERK, JNK, and P38 in hypoxic A549 and H1299 cells transfected with si-Notch4 or si-NC were further measured. Our results demonstrated that Notch4 knockdown in hypoxic A549 and H1299 cells decreased phosphorylation levels of ERK, JNK, and P38 proteins compared with controls (Figure 5A). A rescue experiment in LUAD cells co-treated with Notch4 overexpression plasmid and the specific inhibitors of ERK (U0126), JNK (SP600125), and p38 (SB203580) was performed to evaluate cell proliferation, migration and apoptosis. Our results demonstrated that Notch4 overexpression promoted cell proliferation, migration and inhibited apoptosis, which were partly abolished by co-treatment with the inhibitors (Figures 5B–D, Figure 6). Co-immunoprecipitation assay further revealed a binding interaction between Notch4 and ERK/JNK/P38 in A549 cells (Figure 7). The above results indicate that Notch4 regulates the proliferation, apoptosis, and migration of A549 and H1299 cells via activating ERK, JNK, and P38 phosphorylation.

## Discussion

**FIGURE 8:**
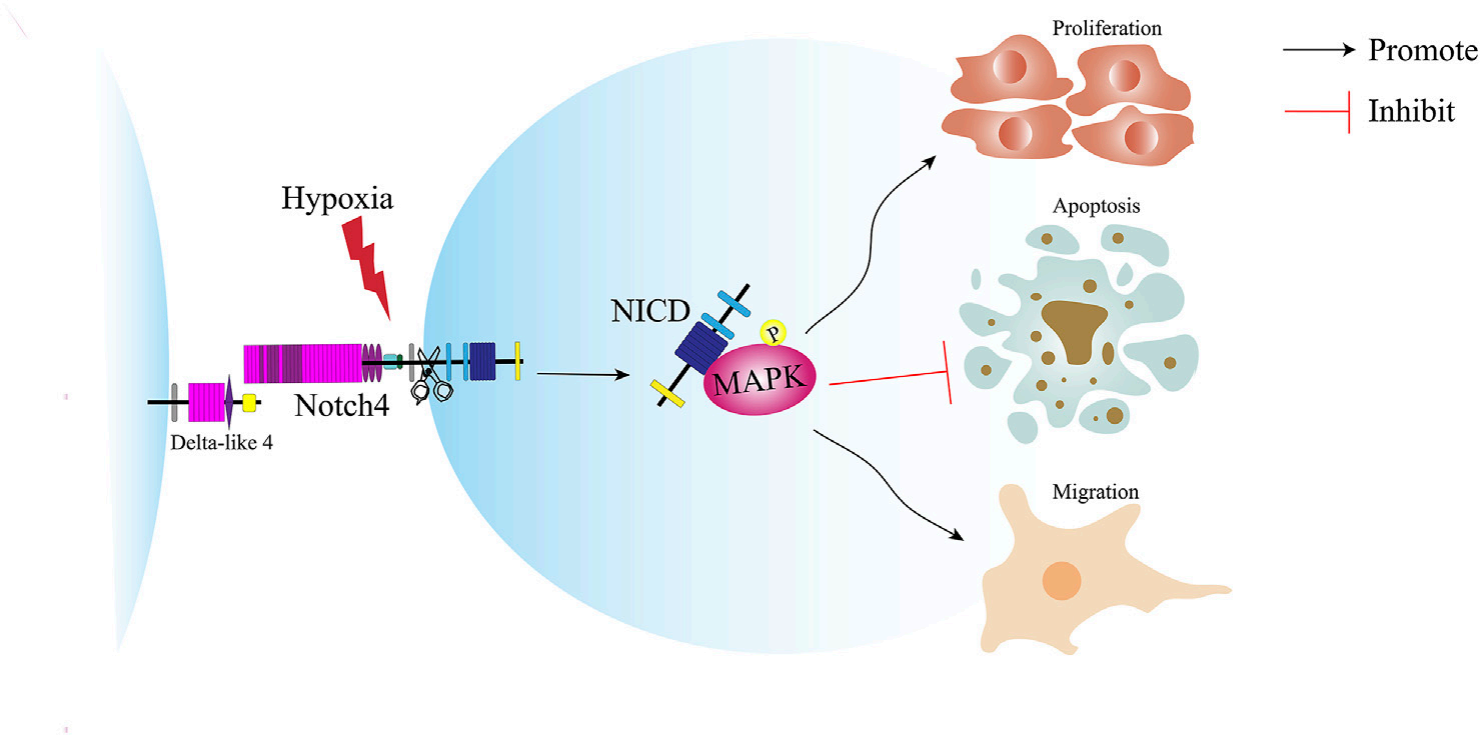
Schematic illustration of the role of Notch4 in the proliferation, apoptosis and migration of LUAD cells. Hypoxia induces the expression of Notch4. Increased level of Notch4 promotes cell proliferation, migration, and apoptosis resistance in A549 and H1299 cells. Hypoxia activates Notch4 via ERK/JNK/P38 MAPK signaling pathways to promote lung adenocarcinoma progression and metastasis. LUAD, lung adenocarcinoma; ERK, extracellular signal-regulated kinase; JNK, c-Jun N-terminal kinase; MAPK, mitogen-activated protein kinase.

In this study, we reveal a novel molecular mechanism underlying the pathogenesis of LUAD and provide a new target for the treatment of LUAD (Figure 8). Notch4 is upregulated in A549 and H1299 cells and human LUAD tissues compared with controls. Hypoxia increases the expression of Notch4 in A549 and H1299 cells, which promotes cell proliferation and migration and inhibits cell apoptosis via the ERK/JNK/P38 MAPK pathway. *In vivo* study shows that Notch4 promotes the progression and metastasis of LUAD.

Notch receptors (Notch1-4) are important for cell fate and function (Zanotti and Canalis, 2016). Previous studies suggest that Notch receptors play a key role in carcinogenesis including inhibition of cell differentiation and apoptosis, and promotion of cell proliferation (Dang et al., 2000; Yeh et al., 2009; Domingo-Domenech et al., 2012). These findings imply that blocking the Notch pathway may improve the prognosis of cancer patients. A monoclonal antibody OMP-59R5 that selectively targeting Notch2 and Notch3 can inhibit xenograft tumor growth (Yen et al., 2015). Hypoxia is a pivotal initiator of tumor growth and metastasis. A nano-scaled polydiaminopyridine nanoparticles doped with iron ions and conjugated with hyaluronic acid has been developed for targeted and oxygen-evolving phototherapy of tumor (Shu et al., 2021). Hypoxia-inducible factor-1α (HIF-1α) activates the transcription of genes involved in cell proliferation and metastasis in cancers (Krishnamachary et al., 2003; Pennacchietti et al., 2003). HIF-1α upregulated the expression of Notch1, Notch3 and Notch4 via binding to the hypoxia response elements in their promoter regions in hepatocellular carcinoma cell lines (Yang et al., 2017). Notch signaling has been shown to mediate the effect of hypoxia on cancer progression and metastasis, such as cervical, colon, glioma, and ovarian cancer (Sahlgren et al., 2008; Irshad et al., 2015; Landor and Lendahl, 2017). However, the role of Notch4 as an oncogene in lung cancer under hypoxia has not been clarified. We thus explored the role of Notch4 in LUAD cell lines by siRNA against Notch4 under hypoxia. Our findings demonstrate that knockdown of Notch4 partly abrogate hypoxia-induced excessive cells proliferation and migration, and apoptosis resistance of A549 and H1299 cells. Targeting Notch4 may provide potential benefits for alternative therapeutic strategies for LUAD.

A defining hallmark of cancer is sustained cell proliferation involving various extracellular and intracellular signaling (Feitelson et al., 2015). Survivin, as a member of the inhibitor of the apoptosis protein family (Ambrosini et al., 1997), promotes tumorigenesis by inhibiting cell apoptosis and promoting cell mitosis (Li et al., 1998). Survivin is overexpressed in a wide range of cancers (Kanwar et al., 2013) and associated with the poor clinical outcomes of patients (Rödel et al., 2012). In this study, we found that hypoxia increased the protein expression of survivin in A549 and H1299 cells, which were downregulated by siRNA against Notch4.

Apoptosis is the most widely studied form of programmed cell death. Apoptosis resistance has been linked to oncogenesis and cancer progression in various types of human cancers (Thompson, 1995). The Bcl-2 family is the best characterized protein family consisting of anti-apoptotic and pro-apoptotic members, such as Bcl-2 and Bax (Martinou and Youle, 2011). The ratio of Bax/Bcl-2 determines the occurrence and degree of cell apoptosis (Ye et al., 2019). Our findings showed that hypoxia inhibited A549 and H1299 cells apoptosis and downregulated the ratio of Bax/Bcl-2, which were partly reversed by siRNA against Notch4.

Tumor metastasis is a complex process that accounts for the majority of cancer-attributed deaths (Lambert et al., 2017). Matrix metalloproteinase (MMP) family is responsible for the extracellular matrix degradation involved in cancer progression (Rao et al., 2005). MMP2 and MMP9 are recognized as two major enzymes in the degradation of type Ⅳ collagen (Stamenkovic, 2003) and correlate with an invasive phenotype of cancer cells (Vihinen and Kähäri, 2002). Previous studies have demonstrated a critical role of MMP9 in the progression of lung cancer and an association between MMP9 and poor survival in patients (Kodate et al., 1997; Suzuki et al., 1998). In this study, hypoxia promoted A549 and H1299 cells migration and increased the protein levels of MMP9, which were both partly reversed by siRNA against Notch4.

The canonical Notch signaling is initiated by ligand-induced cleavage of Notch receptor, followed by intracellular domain translocating into the nucleus and forming a transcriptional activator to activate target genes transcription (Aggarwal et al., 2021; Sprinzak and Blacklow, 2021). Recent evidences indicate that non-canonical signaling is important in oncogenesis via the interaction with PI3K, mTORC2, AKT, MAPK pathway, and HIF-1α in the cytoplasm and nuclear (Ayaz and Osborne, 2014; Li et al., 2016; Zou et al., 2019; Qin et al., 2020). The MAPK pathway is activated in lung cancer and plays a critical role in the development and progression of cancer (Fu et al., 2021; Hu et al., 2021; Jain et al., 2021; Shin et al., 2021). Our results showed that Notch4 silencing downregulated phosphorylation of ERK, JNK, and P38. Meanwhile, Notch4 overexpression increased ERK, JNK, and P38 phosphorylation in A549 and H1299 cells, which was partly abrogated by the specific inhibitors U0126 (Zhong et al., 2021), SP600125 (Zhang et al., 2021), and SB203580 (Kwak et al., 2021). Notably, the increased cell proliferation and migration and inhibited cell apoptosis induced by Notch4 overexpression in A549 and H1299 cells were prevented by the co-administration of U0126, SP600125, or SB203580. Co-immunoprecipitation assay further confirmed the interaction between Notch4 and ERK/JNK/P38. Above all, these results suggest that Notch4 activates the phosphorylation of ERK, JNK, and P38, resulting in increased cells proliferation and migration and inhibited cells apoptosis in LUAD cells.

Cell-to-cell contact is indispensable in ligand-mediated Notch activation, and it is not restricted to the same cell type. Cross-talk between inflammatory cells in the tumor microenvironment and cancer cells plays an essential role in the growth and progression of cancer. Emerging data showed a potential involvement of the Notch pathway in the biology of myeloid-derived suppressor cells, a subset mostly related to the suppression of immune responses in cancer (Grazioli et al., 2017). In addition, a previous study also revealed that activation of Notch1 was enhanced in non-small cell lung cancer cells cultured with *Delta-like 4*-expressing endothelial cells, thus inhibiting the proliferation of non-small cell lung cancer cells and tumor formation (Ding et al., 2012). Here, we have performed immunohistochemistry staining of Notch4 in the lung adenocarcinoma tissues and adjacent non-malignant lung tissues from lung adenocarcinoma patients. Our results showed that the expression of Notch4 increased both in the parenchymal and stroma of tumors compared with normal tissues (Supplementary Figure S5), which indicated that the regulatory effect of Notch4 signal on the proliferation, migration, and apoptosis of lung cancer cells may depend on the interaction between cancer cells to cancer cells or cancer cells to the surrounding cells.

In conclusion, this study demonstrates a critical role of the Notch4-ERK/JNK/P38 MAPK axis in the effect of hypoxia on the progression and metastasis of LUAD and provides a novel potential therapeutic target for LUAD.

## Data Availability

The original contributions presented in the study are included in the article/Supplementary Material, further inquiries can be directed to the corresponding author.

## Abbreviations

LUAD, lung adenocarcinoma; ERK, extracellular signal-regulated kinase; JNK, c-Jun N-terminal kinase; MAPK, mitogen-activated protein kinase; siRNA, small interference RNA; PCNA, polyclonal antibodies against proliferating cell nuclear antigen; Bcl-2, B cell leukemia/lymphoma 2; Bax, BCL2 associated X; MMP, matrix metallopeptidase; IgG, immunoglobulin G; HBE, human bronchial epithelial.
