# Supplementary material for: Hypoxia Activates Notch4 via ERK/JNK/P38 MAPK Signaling Pathways to Promote Lung Adenocarcinoma Progression and Metastasis
Source: Front Cell Dev Biol. 2021 Dec 20;9:780121. doi: 10.3389/fcell.2021.780121 (PMC8721100; doi:10.3389/fcell.2021.780121)
Supplement: Supplementary file 1 [file DataSheet1.docx]

**Hypoxia activates Notch4 via ERK/JNK/P38 MAPK signaling pathways to promote lung adenocarcinoma progression and metastasis**

Xiaochen Li^*1,2^, Xiaopei Cao^*3^, Hanqiu Zhao^1,2^, Mingzhou Guo^1,2^, Xiaoyu Fang^1,2^, Ke Li^1,2^, Lu Qin^1,2^, Yuanzhou He^1,2^, Xiansheng Liu^†1,2^.

^1^Department of Pulmonary and Critical Care Medicine, Tongji Hospital, Tongji Medical College, Huazhong University of Science and Technology, Wuhan, China.

^2^Key Laboratory of Respiratory Diseases, National Ministry of Health of the People's Republic of China and National Clinical Research Center for Respiratory Disease, Wuhan, China.

^3^Department of Pediatrics, Tongji Hospital, Tongji Medical College, Huazhong University of Science and Technology, Wuhan, China.

^*^Contributed equally

^†^Corresponding author

**Running title:** Notch4 contributes to lung adenocarcinoma

**Correspondence to:** Prof. Xiansheng Liu, Tongji Hospital, 1095 Jiefang Avenue, Wuhan 430030, China, E-mail: doctorliu69@126.com

**Supplement Figure**

**
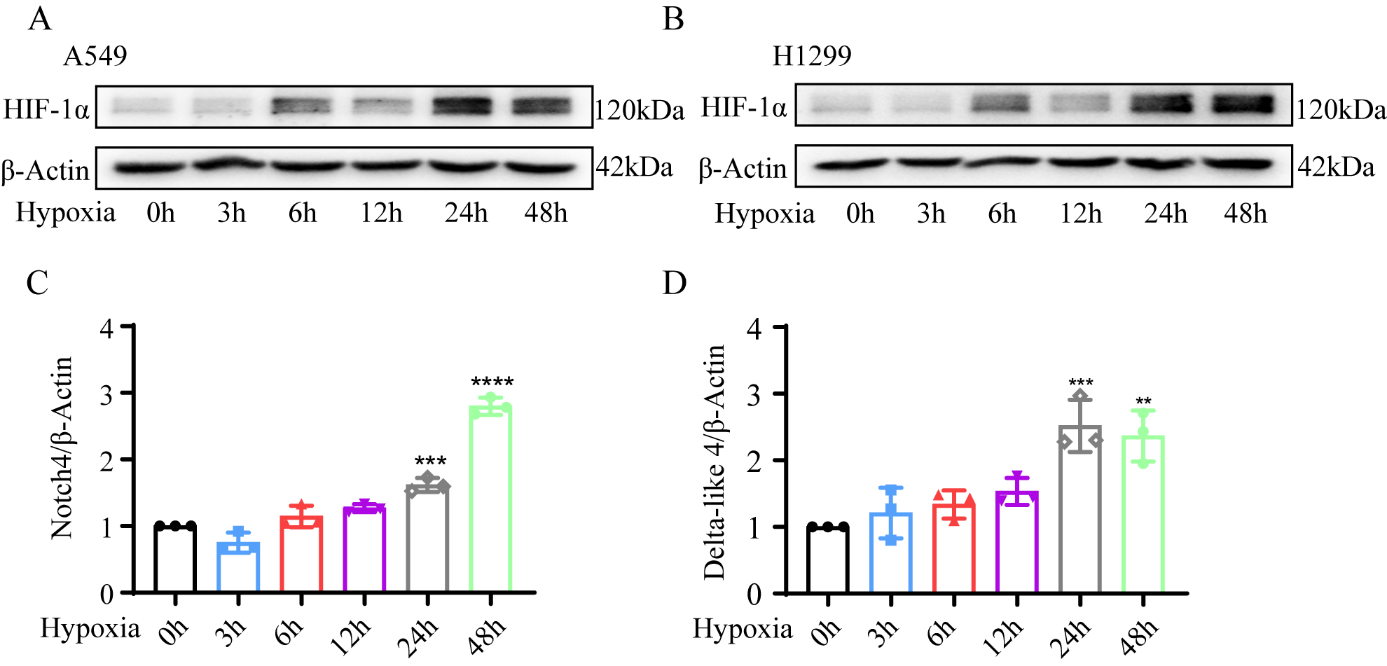
**

**Figure S1. The mRNA levels of Notch4 and Delta-like 4 in H1299 cells exposed to hypoxia.**

**(A, B)** The protein level of HIF-1α in A549 and H1299 cells exposed to hypoxia for indicate time. **(C**) The mRNA level of Notch4 in H1299 cells exposed to hypoxia for indicate time. (n=3). **(D)** The mRNA level of *Delta-like 4* in H1299 cells exposed to hypoxia for indicate time. (n=3). Data were presented as means ± SD. ^**^*P*＜0.01, ^***^*P*＜0.001, ^****^*P*＜0.0001.

**Supplement Figure**

**
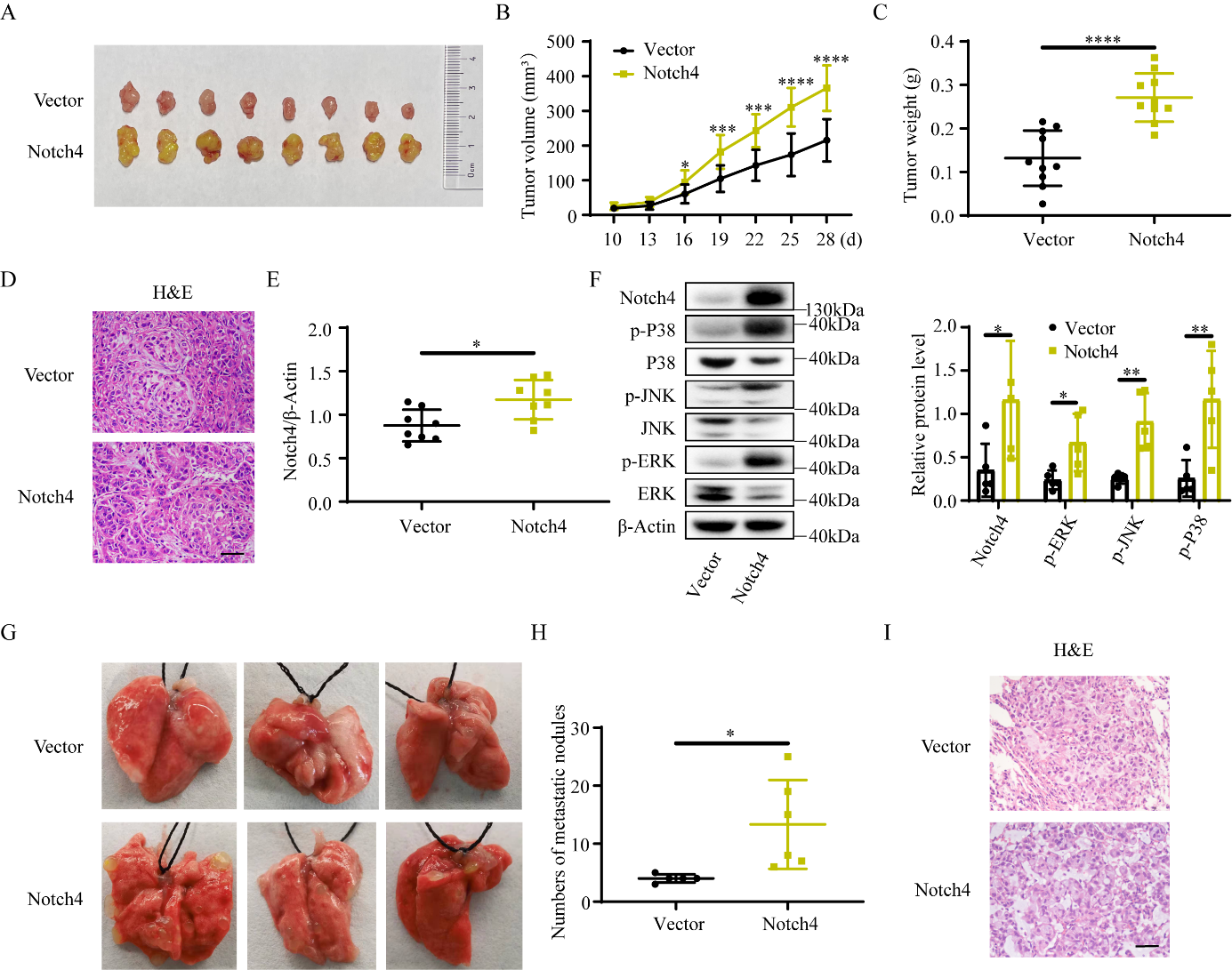
**

**Figure S2. Notch4 facilitated xenograft tumor growth and metastasis.** (A-F) Xenograft tumor growth experiments were performed in nude mice with A549-vector and A549-Notch4 stable cells. **(A)** Representative images of subcutaneous tumor dissected from the nude mice were presented. **(B)** Subcutaneous tumor growth curves of the nude mice were presented. (n=10). **(C)** Subcutaneous tumor weights were presented. (n=10). **(D)** Representative H&E staining images in sections of tumor were presented. Magnification, x200; Bar, 50μm. **(E)** mRNA expression of Notch4 in xenograft tumor. (n=8). **(F)** Protein expression of Notch4, p38, p-P38, JNK, p-JNK, ERK, p-ERK in xenograft tumor. (n=5). (G-I) Lung metastasis experiments were performed in nude mice with A549-control and A549-shNotch4 stable cells. **(G)** Representative images of lung metastases were presented. **(H)** Numbers of visible metastatic nodules in the lungs of mice were counted. (n=5-6). **(I)** Representative H&E staining images in sections of lung tissues were presented. Magnification, x200; Bar, 50μm. Data were presented as means ± SD. ^*^*P*＜0.05, ^**^*P*＜0.01, ^***^*P*＜0.001, ^****^*P*＜0.0001.

**Supplement Figure**


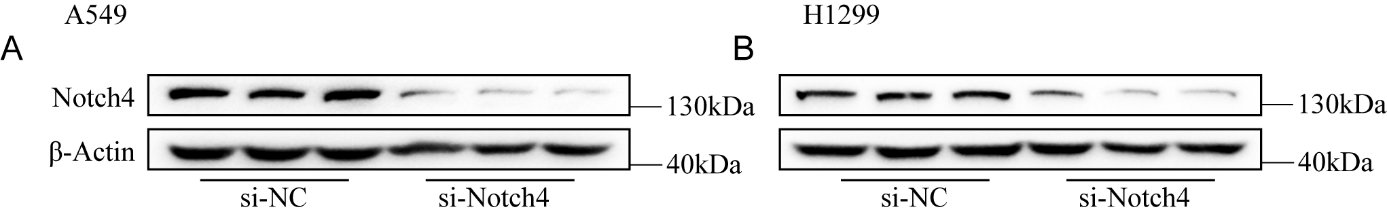


**Figure S3. Notch4 was knocked down in A549 and H1299 cells transfected with siRNA against Notch4.** **(A)** Protein level of Notch4 in A549 cells transfected with the siRNA against Notch4 or negative control siRNA. **(B)** Protein level of Notch4 in H1299 cells transfected with the siRNA against Notch4 or negative control siRNA. si-NC, negative control short interfering RNAs (siRNA); si-Notch4, the siRNA against Notch4.

**Supplement Figure**


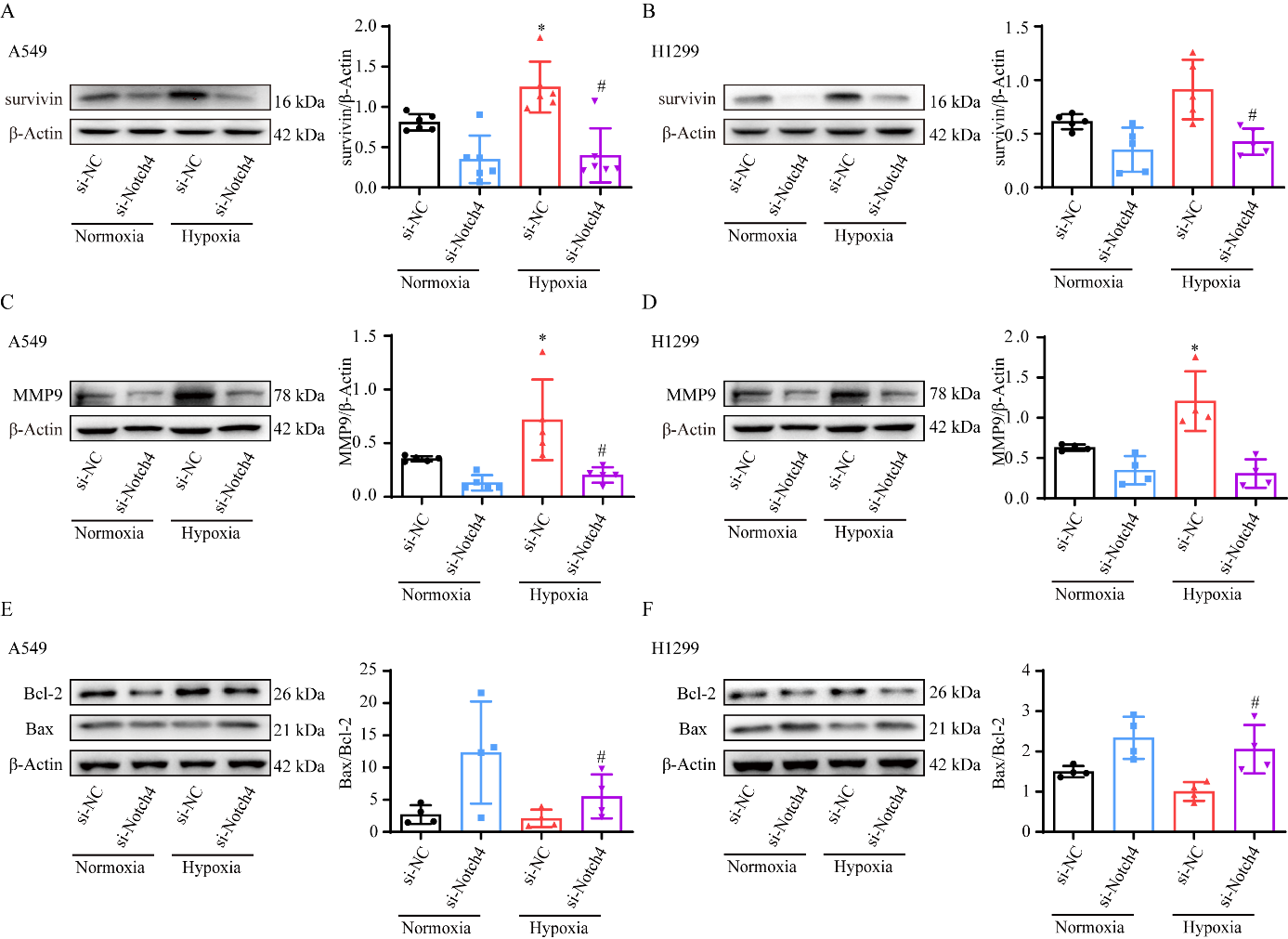


**Figure S4. The regulation of Notch4 on protein levels associated with proliferation, apoptosis and migration.** **(A, B)** Protein expression of survivin in A549 and H1299 cells. (n=5). **(C, D)** Protein expression of MMP9 in A549 and H1299 cells. (n=4). **(E, F)** Protein expression of Bax, Bcl-2 in A549 and H1299 cells. (n=4). Data were presented as means ± SD. ^*^*P*＜0.05, comparison with normoxic cells treated with si-NC; ^#^*P*＜0.05, comparison with hypoxic cells treated with si-NC. si-NC, negative control short interfering RNAs (siRNA); si-Notch4, the siRNA against Notch4.

**Supplement Figure**


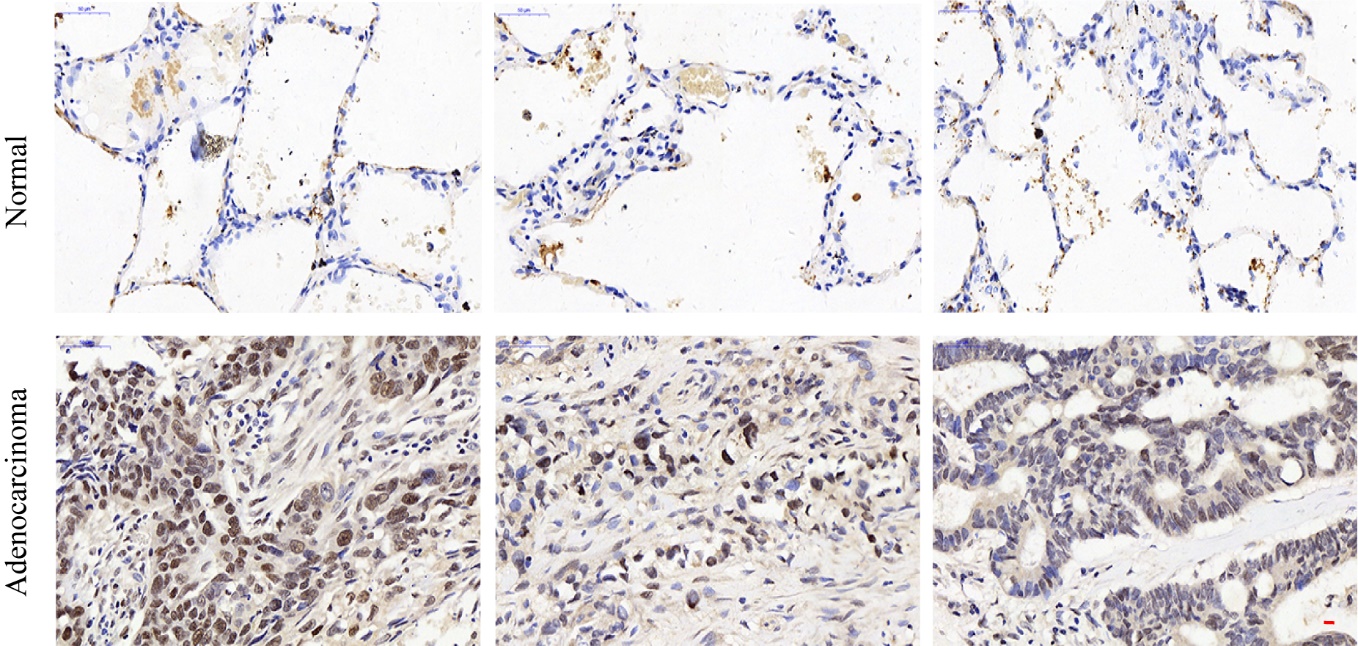


**Figure S5. Notch4 is upregulated in LUAD tissues compared with adjacent normal tissues.** Immunohistochemistry staining of Notch4 in LUAD tissues and corresponding normal tissues. Magnification, x400; Bar, 20μm.
